# A Single Active-Site Mutagenesis Confers Enhanced Activity and/or Changed Product Distribution to a Pentalenene Synthase from *Streptomyces* sp. PSKA01

**DOI:** 10.3390/bioengineering10030392

**Published:** 2023-03-22

**Authors:** Hongshuang Liu, Senbiao Fang, Lin Zhao, Xiao Men, Haibo Zhang

**Affiliations:** 1 State Key Laboratory of Bio-Based Material and Green Papermaking, School of Bioengineering, Qilu University of Technology, Shandong Academy of Sciences, Jinan 250316, China; 2 CAS Key Laboratory of Biobased Materials, Qingdao Institute of Bioenergy and Bioprocess Technology, Chinese Academy of Sciences, Qingdao 266101, China; 3 Shandong Energy Institute, Qingdao 266101, China

**Keywords:** pentalenene, pentalenene synthase, random mutagenesis, site-saturation mutagenesis, molecular docking, molecular dynamics simulations

## Abstract

Pentalenene is a ternary cyclic sesquiterpene formed via the ionization and cyclization of farnesyl pyrophosphate (FPP), which is catalyzed by pentalenene synthase (PentS). To better understand the cyclization reactions, it is necessary to identify more key sites and elucidate their roles in terms of catalytic activity and product specificity control. Previous studies primarily relied on the crystal structure of PentS to analyze and verify critical active sites in the active cavity, while this study started with the function of PentS and screened a novel key site through random mutagenesis. In this study, we constructed a pentalenene synthetic pathway in *E. coli* BL21(DE3) and generated PentS variants with random mutations to construct a mutant library. A mutant, PentS-13, with a varied product diversity, was obtained through shake-flask fermentation and product identification. After sequencing and the functional verification of the mutation sites, it was found that T182A, located in the G2 helix, was responsible for the phenotype of PentS-13. The site-saturation mutagenesis of T182 demonstrated that mutations at this site not only affected the solubility and activity of the enzyme but also affected the specificity of the product. The other products were generated through different routes and via different carbocation intermediates, indicating that the 182 active site is crucial for PentS to stabilize and guide the regioselectivity of carbocations. Molecular docking and molecular dynamics simulations suggested that these mutations may induce changes in the shape and volume of the active cavity and disturb hydrophobic/polar interactions that were sufficient to reposition reactive intermediates for alternative reaction pathways. This article provides rational explanations for these findings, which may generally allow for the protein engineering of other terpene synthases to improve their catalytic efficiency or modify their specificities.

## 1. Introduction

Sesquiterpenes are natural terpenoids with 15 carbon atoms, which are derived from the assembly of three isoprene units [1]. They represent the largest subgroup of terpenoids in terms of the number and type of structural skeleton. Some sesquiterpenes have fragrant odors and a diversity of biological activities, including antioxidant, anti-cancer, anti-inflammatory, and anti-microbial activities, and have been widely used in food, feed, daily chemical, pharmaceutical, energy, and other fields, exhibiting great economic value and broad market prospects [1]. Pentalenene is an angular triquinane (containing three fused five-membered rings) sesquiterpene produced by a variety of *Streptomyces* species, which are solid-welling saprophytic bacterium and antibiotic producers [2,3,4]. Based on calculation, the hydrogenated product of pentalenene was found to have comparable combustion energy to the commercial jet fuel A-1, and it can thus be used as a jet fuel blending agent [5]. Pentalenene is the precursor of antibiotic pentalenolactone, which exerts its antimicrobial activity by the selective irreversible inhibition of glyceraldehyde-3-phosphate dehydrogenase [6,7].

Pentalenene is synthesized by pentalenene synthase (PentS), which is a classic sesquiterpene synthase that has attracted sustained research interest. PentS was first isolated and characterized from *Streptomyces* sp. UC5319 in 1987 [8]. The analysis of its crystal structure revealed that the enzyme was a 38 kDa monomer consisting of 11 α helices (designated A to K) [9,10]. Helix B, C, G, H, and K formed an active cavity approximately 15 Å deep and 9 Å wide [10]. PentS belongs to the class I terpenoid cyclases and contains conserved Asp-rich (DD××D) and NSE/DTE motifs for binding of the diphosphate moiety of the substrate along with three Mg^2+^ ions [11]. Site-directed mutagenesis was performed to understand the structure–function relationship of PentS, including the mutation of the conserved domain, and the replacement of aromatic and basic amino acid residues in the active cavity. For example, replacing the aspartic acid of the aspartate-rich domain with glutamate resulted in decreased catalytic efficiency (*k_cat_*/*K_m_*) and the formation of varying proportions of germacrene A, which were the results of greater steric and conformational freedom in the binding cavity of the carbonium ion intermediates [11]. When the aromatic residue F76 in the active cavity was mutated to tryptophan or histidine, the activity decreased by 68% and 92%, respectively. The F76W mutant produced a small amount of another anti-Markovnikov addition product, α-humulene, in addition to pentalenene, indicating that the benzene ring and pyrrole ring of tryptophan may stabilize the carbonium ion at C9 and C10. The F76H mutant generated trace α-humulene and a significant amount of Markovnikov addition product germacrene A, which was due to the resculpting of the active site such that a different conformation of farnesyl pyrophosphate (FPP) was stabilized in the binding cavity [12]. In addition, the only obvious basic residue in the active cavity is H309, which appears to be the ideal site for the deprotonation of carbocation intermediates to produce pentalenene. Unexpectedly, H309A, H309S, H309C, and H309F mutants retained substantial cyclase activity, with relatively minor changes in *k_cat_*/*K_m_* for all but the H309F mutant, which was presumably caused by relaxed control over the substrate FPP and the carbocationic intermediate conformations in the active sites of the various H309 mutants [13]. These findings clarify the relationships between the structure and function of conserved motifs and provide information for elucidating the catalytic mechanism of PentS. However, the PentS variant with enhanced activity was not obtained.

This study aimed to engineer PentS synthase from *Streptomyces* sp. PSKA01 through the combination of random mutagenesis and saturation mutagenesis to explore novel key active sites and elucidate their structure–function relationships. Firstly, we constructed a pentalenene synthetic pathway and a mutant library harboring PentS variants generated by error-prone PCR. Next, shake-flask fermentation and product identification were carried out to isolate mutants with increased activity or varied product diversity. Then, sequencing and mutation site verification were performed to find which mutation was responsible for the phenotype of the desired mutant. The target residue was subjected to site-saturation mutagenesis and the product diversity of the mutants was analyzed. Finally, the mechanism by which a single active site affected both the activity and specificity of PentS was elaborated in detail through molecular docking and molecular dynamics (MD) simulations. This study enriched our knowledge of the structure–function relationship of terpenoid cyclase and provided useful information for engineering other terpene synthases toward enhanced activity and improved specificity.

## 2. Materials and Methods

### 2.1. General Materials and Culture Conditions

The plasmids and strains used in this study are listed in Table 1. *E. coli Trans*1-T1 was used for the construction of plasmids. *E. coli* BL21(DE3) was used as the host for protein expression and fermentation. During plasmid construction and seed culture preparation, *E. coli* strains were routinely cultured at 37 °C on LB agar plates or in LB liquid medium with appropriate antibiotics. A modified M9 medium described in a previous work was used for shake-flask fermentation [14].

### 2.2. Plasmid Construction and Site-Directed Mutagenesis

Primers used for plasmid construction and site-directed mutagenesis are listed in Table S1. The PentS gene (GenBank accession no. WP_186284259.1) was codon-optimized according to the codon bias of *E. coli* BL21(DE3) and then synthesized by BGI Tech Solutions Co., Ltd. (Beijing, China). *PentS* was amplified by PCR and inserted into the vector pET28a containing *ispA* and *IDI* using a ClonExpress^®^ II One Step Cloning Kit (Vazyme, Nanjing, China) to generate the pE plasmid. Site-directed mutations were introduced to the Y150, T182, F192, and Q209 positions of the *PentS* gene through whole-plasmid PCR amplification using pE as a template. PCR products were digested by *Dpn* I to remove the template plasmid and then transformed into *E. coli Trans*1-T1 and selected on LB agar plates containing kanamycin (50 μg/mL).

### 2.3. PentS Mutant Library Construction and Screening

The PentS mutant library was constructed by random mutagenesis using a Diversify^®^ PCR Random Mutagenesis Kit (Takara) [16]. PentS was amplified with primers erPCR-F and erPCR-R (Table S1) at a mutation rate of 2.3 per 1000 bp. PentS fragments with random mutations were ligated into pET28a containing *ispA* and *IDI* genes using a ClonExpress^®^ II One Step Cloning Kit (Vazyme) and then co-transformed with pT into EC1 (*E. coli* BL21(DE3) containing pA) competent cells and selected on LB agar plates containing chloramphenicol (34 μg/mL), ampicillin (100 μg/mL), and kanamycin (50 μg/mL). Fermentation systems of 15 mL and 50 mL were used for preliminary screening and secondary screening, respectively, to obtain mutants with desired phenotypes.

### 2.4. Shake-Flask Fermentation

The engineered strains EC2 (*E. coli* BL21(DE3) harboring pA, pT, and pE) were cultivated in a 250 mL baffled shake flask containing 50 mL fermentation medium at 37 °C with shaking at 200 rpm. When the OD_600_ reached 0.6–0.9, IPTG was added to the culture to a final concentration of 0.1 mM, and 20% (*v*/*v*) decane was overlaid on the culture for in situ recovery of the products. The cultures were further incubated at 30 °C and 200 rpm for 48 h. The fermentation broth was centrifuged, and the decane layer was taken and analyzed by GC or GC–MS.

### 2.5. Qualification and Quantification of Terpenoids

Fermentation samples were qualitatively detected by GC–MS (Agilent, 7890A-5975C) equipped with a DB-5MS column (30 m × 0.25 mm × 0.25 µm). The flow rate of the carrier gas (Helium) was 1 mL/min. The oven temperature was set at 80 °C for 1 min, increased to 250 °C at a rate of 10 °C/min, held at 250 °C for 1 min, and then increased to 300 °C at a rate of 30 °C/min and held at 300 °C for 1 min. The injector and detector were maintained at 260 °C and 300 °C, respectively. Mass spectrum conditions: EI ionization source; electron energy 70 eV; ion source temperature 230 °C; quadrupole temperature 150 °C; scanning range 40~400 *m*/*z*. Compounds with peak areas >1% of the total peak area in the chromatogram were analyzed. Terpenoids were identified by comparing them with mass spectra data from the NIST08 library.

Fermentation samples were quantitatively detected by GC (SHIMADZU GC-2014) equipped with a hydrogen flame ionization detector (FID) and a DB-5MS column (30 m × 0.25 mm × 0.25 µm). The GC conditions were identical to those used in the GC–MS analysis. β-Caryophyllene was used as an internal standard for the quantification of pentalenene [5]. The products were quantified by the integration of peak areas from the gas chromatography trace using SHIMADZU LabSolutions software and expressed as a percentage of the total products [17]. Data are given as means of three replicates.

### 2.6. Protein Solubility Detection

*E. coli* BL21(DE3) containing the plasmid pE was induced by the addition of 0.1 mM IPTG and incubated at 30 °C and 200 rpm for 18 h. Cells were collected by centrifugation, and the pellet was resuspended in PBS. A small amount of suspension was mixed with SDS-PAGE sample buffer and boiled for 5 min, which was designated as whole cell lysate. The rest of the suspension was ultrasonically broken. The cell lysate was further separated by centrifugation at 16,000× *g* for 15 min at 4 °C. The supernatant containing the soluble proteins was mixed with SDS-PAGE sample buffer and boiled for 5 min. Finally, the same volume of whole-cell lysate samples or soluble fractions was loaded on the protein gel for detection.

### 2.7. Homology Modeling and Molecular Docking

The homology modeling for PentS was conducted using Modeller software [18]. Multiple-sequence alignment was generated by CLUSTALW and ESPript 3.0 [19]. The crystal structure of PenA (PDB: 6wkd) [12], which shares 92.9% of its sequence identity with PentS, was selected as a modeling template for the 3D structural analysis of PentS. A > 10 ns position-restrained MD simulation was performed to eliminate the steric conflicts of the modeled protein.

The molecular docking process was performed by Autodock 4.2 to predict the binding models of PentS with FPP [20]. Before docking, the ligand FPP was sketched using ChemDraw 2014 and imported into the molecular operating environment (MOE) software to conduct the 3D protonation and energy minimization. Then, the chemical structure of FPP was saved in the PDB format. The docking protocol began with a rigid-body docking in which the binding pocket was strictly positioned a 10 Å distance away from the ligand FPP. Metal ions of Mg^2+^ and residues forming the binding pocket were both considered in the grid-docking box. Pose clustering was employed and a maximum number of 100 conformations were considered; finally, the docking pose with the lowest binding free energy was selected as the PentS–FPP complex.

### 2.8. Mutant Construction and Molecular Dynamics Simulations

The mutation process of structures of the 19 site-saturation mutants was executed based on the wild-type PentS–FPP complex with the Mutagenesis Wizard tool of PyMol software [21]. At least 10 ns MD simulations for each mutant were performed using Amber20 software to gain insight into the thermodynamic and structural details of mutation-linked tertiary changes [22].

The protein–ligand system was solvated into a model with one cubic box of TIP3P water (100 × 100 × 100 Å) allowing a minimum of 10 Å distance from heavy atoms on protein to each side of the water box. The GAFF and AMBER99SB force field parameters for the ligand and protein were parameterized. The periodic boundary conditions were implemented, and each amino was assigned as the standard ionization state at a pH = 7.0 physiological condition. Na^+^ ions were added by using the Monte Carlo ion-placing method to neutralize the whole protein–ligand system. Subsequently, the constant 1000 kJ/mol force restrained MD simulations for all heavy atoms of protein and the pyrophosphate head group were conducted. The FPP carbon chain was freely optimized with no force restrained within simulations. Initial geometry optimizations of each system were conducted using 5000 iterations (5 ps) with the steepest descent algorithm. Two-staged equilibration steps conditioned for 100,000 iterations (100 ps) under constant NVT (number of particles, volume, and temperature) and constant NPT (number of particles, pressure, and temperature) ensembles were proceeded to minimize the system. The root mean square deviation (RMSD) of all heavy atoms for each single mutant was verified until the MD simulations reached an equilibrium state. The binding model between each PentS and FPP was extracted for detailed analysis. The binding free energies of the protein–ligand complex were calculated using the MMPBSA module, and the average structure was extracted for conformational analysis. The average coordinate of heavy atoms from residues forming the binding pocket was defined as the geometry center.

## 3. Results

### 3.1. Screening of PentS Mutant Library and Mutation Identification

**Figure 1:**
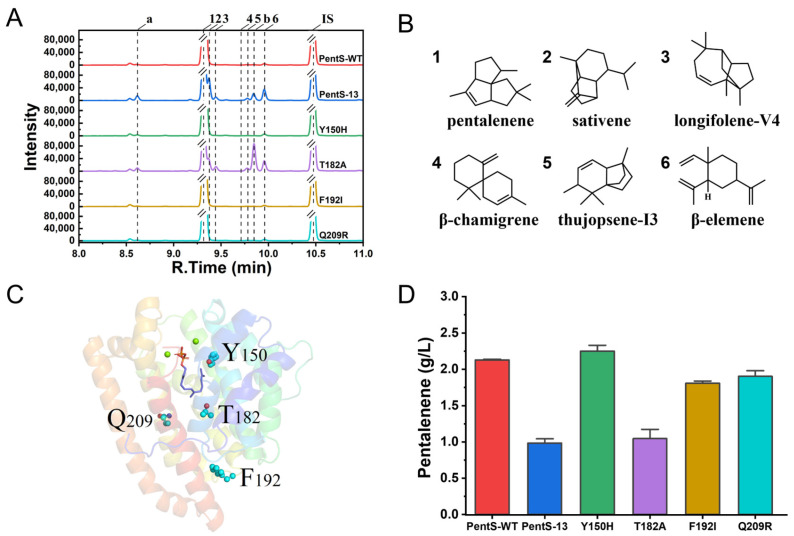
Product spectrum and pentalenene production of wild type and mutants of PentS. (**A**) Comparison of gas chromatogram between wild type and mutants. Peaks **a** and **b** indicate unidentified hydrocarbons with *m*/*z* 204. **1**–**6** were tentatively predicted as pentalenene, sativene, longifolene-V4, β-chamigrene, thujopsene-I3, and β-elemene, respectively. IS, the internal standard β-caryophyllene. (**B**) Chemical structures of sesquiterpenes detected. (**C**) Three-dimensional structure of wild-type PentS and location of the four mutation sites of PentS-13. (**D**) Pentalenene production of wild-type PentS and the four single-site mutants.

Based on a previously constructed MVA pathway and the introduction of a pentalenene synthase (PentS) from *Streptomyces* sp. PSKA01, a pentalenene synthetic pathway was constructed in *E. coli* BL21(DE3) (Figure S1). The shake-flask fermentation of the strain was performed and the product was analyzed by GC or GC–MS. The results show that pentalenene was the major product, accounting for more than 98% of the total product (Figure 1A). The titer of pentalenene was up to 2.13 g/L, which was much higher than the reported highest pentalenene production in the engineered *E. coli* strain, namely 780.3 mg/L (Table 2) [5]. To explore the new active site and further understand the structure–function relationship of PentS, we introduced random mutation to PentS by error-prone PCR. After screening up to 341 colonies, a mutant PentS-13 was found to have a varied product diversity. While pentalenene (**1**) was still the major product, other products such as sativene (**2**), longifolene-V4 (**3**), β-chamigrene (**4**), thujopsene-I3 (**5**), and β-elemene (**6**) were increased at different degrees (Figure 1A,B and Figure S2). Among them, **2**, **3**, and **4** were new sesquiterpenes that were not found in the products of strains harboring the wild-type PentS. The sequencing results indicate that the mutant had four mutations, Y150H, T182A, F196I, and Q209R, located in the F helix, G2 helix, random coil at the junction of G2 helix and H helix, and H helix, respectively (Figure 1C). To determine which mutation contributed to the changed product diversity, we created single-site mutants of Y150H, T182A, F196I, and Q209R by site-directed mutagenesis, respectively. Compared with the wild type, the pentalenene production of T182A, F192I, and Q209R decreased by 50.9%, 15.1%, and 10.6%, respectively, whereas the pentalenene production of the Y150H mutant did not change significantly (Figure 1D). The T182A single mutant displayed a very similar product profile and titer to the PentS-13 mutant (Figure 1A). Thus, we confirmed that T182A was responsible for the changed production diversity of PentS-13.

### 3.2. Site-Saturation Mutagenesis of Residue T182

To further examine the effect of the 182 position on the enzyme properties, we performed site-saturation mutagenesis at this site. The protein solubility was detected through sodium dodecyl sulfate-polyacrylamide gel electrophoresis (SDS-PAGE). According to the results, the wild type and mutants of PentS had similar expression levels (Figure S3A,B). As shown in Figure S3C,D, almost no soluble proteins of the T182D, T182E, T182N, and T182P variants were observed in the supernatant of the cell lysate. The protein solubility was significantly reduced for the T182G, T182H, T182L, T182Y, and T182Q variants. The soluble protein contents of other mutants were essentially the same as that of the wild type.

**Figure 2:**
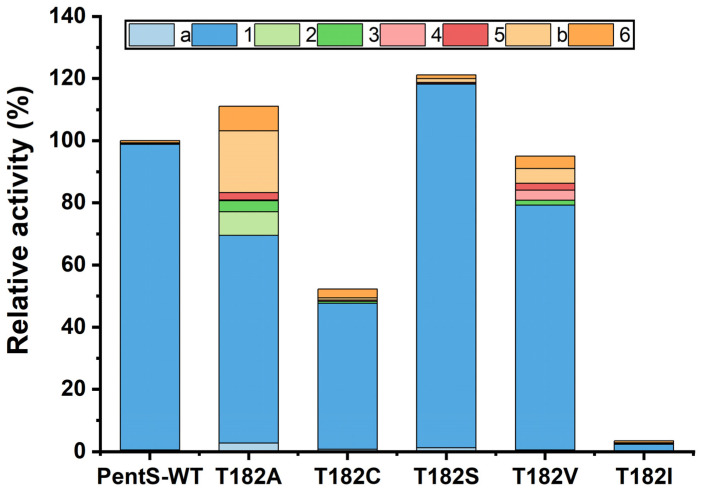
Relative activity and product distribution of wild type and variants of PentS. The relative activity was determined by the peak area of sesquiterpene. The relative activity of wild-type PentS (total sesquiterpene production) is 100%. **a** and **b** indicate unidentified hydrocarbons with m/z 204. **1**–**6** were tentatively predicted as pentalenene, sativene, longifolene-V4, β-chamigrene, thujopsene-I3, and β-elemene, respectively.

Subsequently, we analyzed the relative activities of variants with the same protein solubility. When the T182 residue was substituted by Met, Trp, Phe, or Arg, the activity of PentS was completely lost. As shown in Figure 2, Figure S4, and Table S2, the T182I mutant resulted in a marked decrease in enzyme activity; less than 3% of activity remained. The product distribution of the T182I mutant changed dramatically, with a decreased proportion of pentalenene and increased proportions of other products. The T182A, T182C, T182S, and T182V variants retained substantial cyclase activity. Remarkably, the T182S variant showed both increased total relative activity and pentalenene synthase relative activity (121.1% and 119.0%, respectively) compared with that of the wild type. It also had a similar product profile to the wild-type PentS. The T182A variant exhibited increased total relative activity by 11.1% but decreased pentalenene production along with the changed product distribution. Proportions of products **2**, unknown **b**, and **6** were increased. The total relative activity of the T182V variant was essentially the same as that of the wild type. However, the pentalenene production was reduced by 19.9%, while the proportions of other products such as **3**, **4**, **5**, unknown **b**, and **6** were increased. The T182C variant exhibited both decreased total relative activity by 47.7% and an altered product distribution. Previous studies demonstrated that the pentalenene synthase mutants had other coproducts, such as germacrene A, protoilludene, α-humulene, and β-caryophyllene, besides the main product pentalenene [11,12,13]. Herein, the presence of product **6** is consistent with the previous report, while other products (**2**, **3**, **4**, and **5**) were discovered in this study for the first time.

### 3.3. Synthetic Pathways of Sesquiterpenes in Wild Type and Variants of PentS

**Figure 3:**
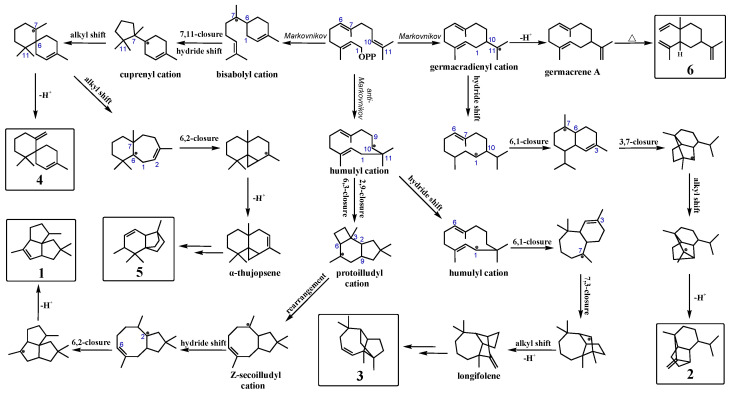
Predicted sesquiterpenes biosynthetic pathways of wild-type PentS and its variants. The products **1** and **3** were pentalenene and longifolene-V4, respectively, which were synthesized through anti-Markovnikov route initialed by 10, 11 double-bond attacked C1 and formed humulyl cation with positive charge on the less-substituted carbon (C10). The products **2** (sativene) and **6** (β-elemene) were generated obeying Markovnikov’s rule initialed by 10, 11 double-bond attacked C1 and formed germacradienyl cation with positive charge on the more highly substituted carbon (C11). The products **4** (β-chamigrene) and **5** (thujopsene-I3) were also generated following Markovnikov’s rule initialed by 6, 7 double-bond attacking of C1 and formed bisabolyl cation with positive charge on the more highly substituted carbon (C7).

Most terpene synthases follow Markovnikov addition reactions to produce high-energy carbonium ion intermediates [12], such as aristolochene synthase [24], bisabolene synthase [25], and guaiene synthase [26]. On the contrary, PentS undergoes an initial anti-Markovnikov addition to form pentalenene. Possible cyclization pathways for wild-type PentS and mutants to generate various sesquiterpenes from FPP are illustrated in Figure 3. The cyclization pathways have been extensively studied using a variety of stereo-specifically labeled forms of FPP [12,27,28]. Products of wild-type PentS and variants were mainly generated through three pathways as follows: (1) **1** and **3** were generated through anti-Markovnikov pathways. First, the pyrophosphate moiety was initially ionized under the action of Mg^2+^, and the C10/C11 double attacked C1 through anti-Markovnikov addition to provide the humulyl cation. Then, the humulyl cation underwent a hydride shift and transannular cyclization to produce the protoilludyl cation. The protoilludyl cation underwent rearrangement to the Z-secoilludyl cation, followed by a hydride shift, transannular cyclization, and deprotonation to form **1** [12,29,30]. Similarly, the humulyl cation was converted to longifolene via hydride shift, cyclization, rearrangement, and deprotonation; finally, longifolene was rearranged by acid-catalyzed reactions to give **3** [31,32,33]. (2) **2** and **6** were formed through Markovnikov pathways. The allylic carbocationic C10 attacked C1 to give the germacradienyl cation. Then, germacradienyl cation underwent a hydride shift, transannular cyclization, and deprotonation to produce **2 [34]**. The germacradienyl cation was converted to **6** via deprotonation and Cope rearrangement [12]. (3) **4** and **5** were also generated through Markovnikov routes. First, the C6-C7 alkene attacked C1 to give bisabolyl cation. Bisabolyl cation underwent 7,11 cyclization and a hydride shift to form the cuprenyl cation. Then, the bisabolyl cation underwent a hydride shift, transannular cyclization, alkyl shift, and deprotonation to give **4 [25]**. Similarly, the cuprenyl cation was converted to α-thujopsene via a hydride shift, cyclization, rearrangement, and deprotonation; finally, α-thujopsene was converted to **5** through a series of reactions [35,36]. The increased proportions of other products in PentS variants indicated that T182 played an important role in guiding the regioselectivity of carboncation intermediates during the cyclization reactions.

### 3.4. Binding Interactions between Wild-Type PentS and FPP

**Figure 4:**
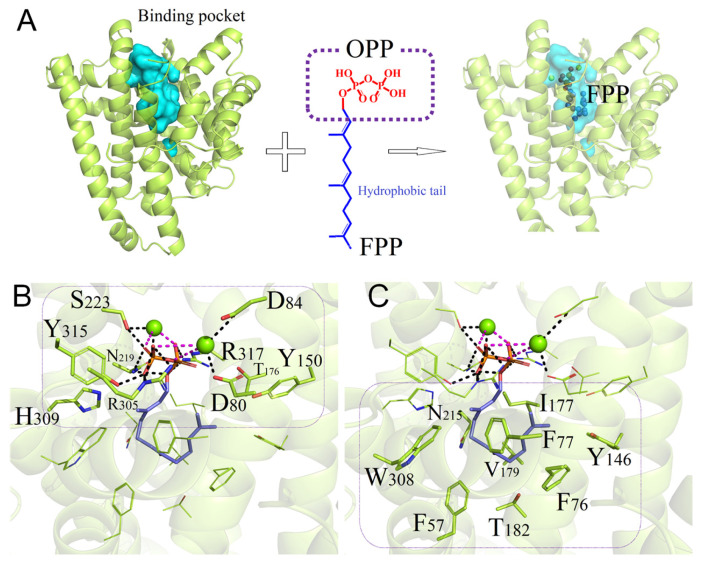
Binding model of the wild-type PentS with ligand FPP. (**A**) Molecular docking analysis of PentS with substrate FPP. The binding cavity was colored in cygan. The ligand FPP consists of two parts: the hydrophilic pyrophosphate head group OPP and the hydrophobic prenyl tail. (**B**) Binding region of PentS cavity with the head group OPP. Representative polar or charged residues are indicated in the rounded rectangle. Polar interaction and metal ion chelation are represented by black dashed lines and fuchsia dashed lines, respectively. (**C**) Binding region of PentS cavity with the hydrophobic prenyl tail. Representative aromatic residues or aliphatic residues are indicated in the rounded rectangle. The residues and FPP are shown as sticks and Mg^2+^ ions are represented as green balls.

To elucidate the structure and correlation with the catalytic activity of PentS, sequence alignment and three-dimensional structure simulation were performed. PentS has the ^80^DD××D/E, ^173^R, ^219^NSE/DTE, and ^314^RY conserved motifs and T182 is not strictly conserved among different *Streptomyces* species (Figure S5). As shown in Figure 4A, the binding pocket of PentS for FPP is an oval-like cavity surrounded by five α-helix bundles. Molecular docking analysis revealed that the substrate FPP can be buried in the binding cavity with −9.19 kcal/mol binding free energy. At the top of the substrate channel, the ligand binds strongly to the Mg^2+^ ions in a bidentate manner through its pyrophosphate moiety. Polar or charged residues, such as D80, D84, N219, S223, R305, H309, Y315, and R317, are required for the binding of Mg^2+^ ions and stabilization of the pyrophosphate leaving group. Salt–bridge interactions are formed of the pyrophosphate moiety with the neighboring charged residues, such as R305, while hydrogen-bond networks exist with polar residues, such as N219, S223, and Y315 (Figure 4B). At the bottom of the binding pocket, nine residues (F57, F76, F77, Y146, I177, V179, T182, N215, and W308) directly participate in forming the catalytic domain of PentS [12,37]. For the benzene rings of F57, F76, and F77 and indole ring of W308, all of them make hydrophobic contact with the prenyl tail of FPP (Figure 4C). According to the physicochemical properties of the binding surface, six residues (F57, F76, F77, I177, V179, and W308) were identified to drive hydrophobic interaction and stabilize the PentS–FPP complex. It is worth noting that the T182 residue containing the weak polar -OH group is located at the bottom of the hydrophobic pocket, exhibiting incompatible properties with the surrounding environment, implying that the residue T182 may play an important role in the substrate binding and/or catalysis process.

### 3.5. Binding Properties of PentS Variants with FPP

Molecular docking and MD simulations were performed to investigate the structures and dynamics of the 10 PentS variant–FPP complexes in the physicochemical environment. The binding free energy and binding pocket volume were calculated for each system. The distance between the atom O4 (oxygen atom connecting two phosphorus atoms in P_2_O_7_^4−^) and the geometric center of the binding pocket (D_O-C_) was monitored to quantify the global conformational change in the variant–FPP complex with respect to the structure of the wild-type system.

As shown in Table 3, in comparison to the wild-type PentS, five T182 mutations own residues with visibly larger sizes (T182F, T182W, T182M, T182K, and T182R) and/or positively charged side chains (T182K and T182R), which would reduce the cavity volume and/or change the electronic properties of the binding cavity, leading to the enlargement of D_O-C_ and increase in binding free energy, finally resulting in a sharp decrease in binding affinity and loss of catalytic activity. A slightly decreased binding cavity volume and increase in both binding free energy and D_O-C_ are observed in variant T182I, indicating that this mutation also seriously affects the binding model of the PentS–FPP complex, and leads to a significant reduction in the product yield. The last four variants, T182C, T182A, T182V, and T182S, have similar or slightly higher binding free energies than that of the wild-type system, implying that the substrate-binding affinity is less affected. The D_O-C_ stays within 4.6~4.7 Å, which is also comparable to the wild-type system. Three of the four variants give a palpable rise in binding pocket volumes. Overall, the above results are consistent with the relative activity analysis (Figure 2), especially the D_O-C_ values, which are negatively correlated with the pentalenene production of the last four variants.

**Figure 5:**
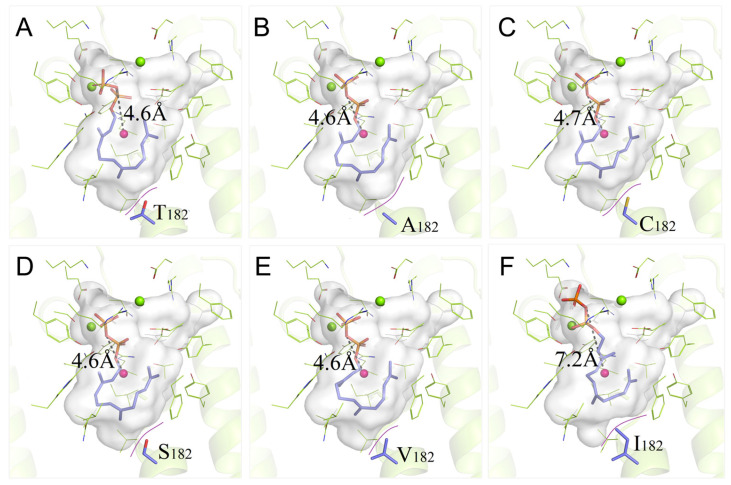
Binding pattern comparisons of PentS–FPP complexes. (**A**–**F**) are the simulation diagrams showing FPP conformations in the active cavities of the wild type (T182), T182A, T182C, T182S, T182V, and T182I variants, respectively. The secondary structures of wild type and variants of PentS are shown as cartoon and lemon colored. The binding cavities are outlined by white surfaces. The residues at position 182 are shown as sticks in which atoms are color-coded as follows: C, blue; N, dark blue; O, red; S, yellow. The geometric center of the binding cavity of the wild-type PentS (red sphere) was used as a comparing template, and the D_O-C_ is marked with black dashed lines.

To further understand the role of the 182nd residue and elucidate the structure–activity relationship of PentS, wild-type PentS together with the 10 variants were conducted for detailed analysis of the binding pattern variations. As shown in Figure 5A, for the wild-type PentS–FPP complex, one close geometric matching identified that the hydrophobic carbon chain tail of FPP curled itself inside the bottom of the binding pocket with a D_O-C_ of 4.6 Å. A structural change in the 182nd position can trigger significant changes in binding models between variants and FPP, and can induce variation in the catalytic activity. Among the 10 variants, T182A, T182C, T182S, and T182V maintain similar conformations with the most approximate D_O-C_ compared with the wild-type PentS–FPP complex (Figure 5B–E). These mutations caused gentle changes in the binding pockets and small fluctuations in the binding free energies, which resulted in changed catalytic activities to varying degrees.

When T182 mutated to I182, the extended side-chain prompted a sharp decrease in the ligand binding space and tended to squeeze the polar head of FPP out of the pocket (see Figure 5F), which enlarged the D_O-C_ and attenuated the binding ability of the T182I mutant. Therefore, the pyrophosphate anion group could not maintain the bidentate manner to the Mg^2+^ ions and led to the almost complete loss of catalytic activity.

More extreme interference occurred in variants harboring charged residues, as the charged residues (K182 and R182) completely destroyed the original physicochemical characteristics in the wild-type PentS–FPP complex. As shown in Figure S6C,E, the positively charged residues (K182 and R182) enhanced the repulsion to the hydrophobic tail of FPP and resulted in increased binding free energies of −8.37 kcal/mol and −6.53 kcal/mol, respectively. Besides the physicochemical properties, the geometric characteristics also play critical roles in maintaining the catalytic activity of PentS. As shown in Figure S6B,D,F, residues with large side-chains (W182, F182, and M182) reduced the volume of the cavity and compelled the pyrophosphate head of FPP out of the binding region. The maximum difference of the D_O-C_ was observed in mutant T182F. FPP could not fold itself properly in the binding cavity of these five variant systems, resulting in significantly increased binding free energies of −8.31~−6.53 kcal/mol (Table 3) and a complete loss of catalytic activity. The above results suggest that altering the volume size or the polarity of the binding pocket had evident effects on the catalytic function of terpenoid synthase.

## 4. Discussion

Pentalenene synthase belongs to the classic class I terpene synthase and was the first enzyme to be identified in prokaryotes [9,38]. The previous studies on the active sites of the pentalenene synthase mainly focused on the conserved motifs, aromatic residues, and charged residues, and most mutants exhibited decreased catalytic activities [11,12,13]. In this study, we found a novel active site of PentS, the T182 residue, through random mutagenesis and mutant screening. Site-saturation mutagenesis at this position revealed that this active site could not only affect the protein solubility but also the product yield and specificity. For example, the substitution of Thr with Ser led to increased production of both the total sesquiterpenes and pentalenene. This mutant was the only variant with higher pentalenene synthase activity compared with the wild type; hence, it provided a clue for engineering other terpene synthases toward enhanced activity and laid the foundation for the commercial production of high-value terpenes. Substitutions of T182 with Ala, Cys, or Val retained substantial cyclase activity. However, these mutations resulted in a decreased production of pentalenene and an increased production of other sesquiterpenes at different degrees. The other sesquiterpenes were generated through different routes and via different carbocation intermediates. These results suggest that the 182 active site is crucial for PentS to stabilize and guide the regioselectivity of carbocations, and that non-aromatic residues, including polar residues, made substantial contributions during the reactions, which contradicts previous opinions according to which the polar residues are not well-suited for stabilizing carbocations [39].

T182 is located in the G helix. The G helix has been reported to play important roles in protecting the substrate carbocation from exposure to the outer environment, and it is involved in regioselectivity and product specificity of terpene synthases. Single or multiple mutations of the key active sites in the G helix of terpene synthases could change the selectivity of products or affect the catalytic activity [38,40,41,42]. For example, the random mutagenesis and rational design of the G helix of the (+)-δ-cadinene synthase resulted in the N304P/L405H mutant, which maintained the cyclase activity and changed its product from (+)-δ-cadinene to germacrene D-4-ol [38]. The investigation of TPS4 and TPS5 from *Zea mays* found that the stereoselectivities of the two terpene synthases could be significantly affected by residue 409 located in a highly conserved kink of the G helix. The change from Gly 409 to Ala resulted in a variant that produced mostly (S)-β-bisabolene with only minor amounts of the bicyclic compounds, sesquithujene and sesquisabinene, which may be due to the fact that the additional methyl group altered the shape of the cavity wall or changed the distance between the F helix and G helix and thus changed the relative rate of the formation of different carbocation intermediates [43]. Srivastava et al. found that mutations in the G helix kink region of germacradien-11-ol synthase resulted in a drastic change in the product profile, with G188A in particular leading to the formation of the complex non-hydroxylated isolepidozene as the major product, which proved that the residue 188 played a key role in the rearrangement of the G helix in active site closure and substrate ionization [41]. Shukal et al. engineered fungal viridiflorol synthase through phylogeny-based mutation combined with random mutagenesis and found that the G-helix-located E267S could enhance the viridiflorol-specific yield of the V314Y-G227C-E267S triple mutant, whereas the E267S single mutant exhibited a significantly reduced viridiflorol yield [42]. The T182 residue presented in this study is not located at a similar position as the above active sites, indicating that it is a novel key active site that has never been characterized before. T182 is located at the lower part of the G2 helix, which is near the bottom of the binding pocket. T182 mutations affected the conformation and hydrophobic/polar properties of the G2 helix. The significantly changed product profiles of T182 mutations further supported that the G helix is an important catalytic feature of terpene synthases that has a profound effect on product specificity.

Molecular docking and MD simulations were employed in elaborating the detailed binding properties and binding models of the wild type and variants of PentS. The calculated binding properties provide useful information for understanding the PentS–FPP complex; the D_O-C_ has a particularly good negative correlation with the catalytic activities, which suggests that these methods are reliable in analyzing the structure of enzyme without crystallization trials. It was revealed that residue at the 182 position could affect both the product yield and specificity by altering the shape and volume of the active cavity and subtly changing the hydrophobic/polar interactions between the variants and FPP. The change in the conformation and properties of the active cavity led to the corresponding change in the conformation of FPP, which resulted in varied binding affinities and catalytic activities [41]. The conformation change in FPP probably influenced the possibility and frequency of other carbon atoms attacking the C1 cation intermediate after FPP ionization and increased the generation of different carbocation intermediates, which contributed to the various product profiles. These results support the opinion that the degree of conformational flexibility of the substrate in the active site of a terpene synthase is a major determinant of product selectivity [44] and proved that the 182 active site is crucial for the catalytic activity and regioselectivity of PentS.

## 5. Conclusions

In this study, PentS from *Streptomyces* sp. PSKA01 was engineered through non-rational design, rational design, and semi-rational design. Through random mutant library construction and screening, a mutant PentS-13 with decreased pentalenene production and increased production of other sesquiterpenes was obtained. Site-directed mutagenesis and functional analysis proved that T182A mutation was responsible for the changed product yield and profile. The site-saturation mutagenesis of T182 demonstrated that residue 182 affected the protein solubility and played important roles in maintaining enzyme activity and product specificity. Molecular modeling helped to illustrate the PentS–FPP complexes and reveal that mutations at the 182 position may induce changes in the cavity shape and volume and disturb hydrophobic/polar interactions sufficiently to reposition reactive intermediates for alternative reaction pathways. This study contributed to the analysis of the stereoselectivity mechanism of terpene synthase and provided theoretical and methodological references for the engineering and structure–function analysis of other terpene synthases. These findings could also be helpful in the generation of high-performance industrial enzymes and strains and boost the quantity and purity in the production of value-added compounds.

## Figures and Tables

**Table 1:** Plasmids and strains used in this study.

Name	Relevant Genotype	Sources
**Plasmids**		
pACYCDuet-1	P15A ori; Cm^R^; P_T7_	Novagen
pTrcHis2B	pBR322 ori; Amp^R^; P_trc_	Invitrogen
pET28a	pBR322 ori, f1 ori; Kan^R^; P_T7_	Novagen
pA	P15A ori; Cm^R^; P_T7_:: *mvaE-mvaS*	[15]
pT	pBR322 ori; Amp^R^; P_trc_:: *ERG12-ERG8-ERG19*	Lab stock
pE	pBR322 ori, f1 ori; Kan^R^; P_T7_:: *PentS* (WT or mutants)*-ispA-IDI*	This work
**Strains**		
*E. coli Trans*1-T1	F^-^ψ80 (*lac*Z)ΔM15Δ*lac*X74 *hsd*R (r_k_^−^,m_k_^+^)Δ*rec*A1398*end*A1t*on*A	TransGen Biotech
*E. coli* BL21(DE3)	*E. coli B dcm ompT hsdS(rB*^−^ *mB* ^−^ *) gal*	TaKaRa
EC1	*E. coli* BL21(DE3) harboring pA	This work
EC2	*E. coli* BL21(DE3) harboring pA, pT and pE	This work

**Table 2:** Comparison of pentalenene production in the engineered strains.

Chassis	Name	Source	Titer (mg/L)	Reference
*Saccharomyces cerevisiae*	PentS	*Streptomyces* sp. UC5319	334.7	[5]
*Xanthophyllomyces dendrorhous*	PPS	*Streptomyces* sp. UC5319	0.25–0.68	[23]
*E. coli*	PentS	*Streptomyces* sp. UC5319	780.3	[5]
*E. coli*	PentS	*Streptomyces* sp. PSKA01	2130	This work

**Table 3:** Binding properties of wild type and variants of PentS with FPP.

Variant	V_VdW_	Polarity	V_Cavity_	∆G	D_O-C_	Variant	V_VdW_	Polarity	V_Cavity_	∆G	D_O-C_
T182	93	P	797.82	−9.19	4.6	T182I	124	H	779.96	−8.67	7.2
T182F	135	H	760.11	−8.31	10.5	T182C	86	P	801.36	−9.06	4.7
T182W	163	H	730.21	−7.30	9.5	T182A	67	H	824.06	−9.10	4.6
T182M	124	H	768.73	−7.56	9.2	T182V	105	H	790.29	−8.93	4.6
T182K	135	C+	756.24	−8.37	9.0	T182S	73	P	815.81	−9.16	4.6
T182R	148	C+	745.56	−6.53	8.5						

VVdW (Å^3^): volume size of each residue; Polarity: polarity of each mutated residue (P: polar; H: hydrophobic; C+: electronic positive charged); V_Cavity_ (Å^3^): volume size of the binding cavity; ∆G (Kcal/mol): binding free energy for each variant and ligand; D_O-C_ (Å): distance between the geometric center of the binding cavity and the O4 atom of the head group P_2_O_7_^4−^.

## Data Availability

Not applicable.

## Supplementary Materials

The following supporting information can be downloaded at: https://www.mdpi.com/article/10.3390/bioengineering10030392/s1, Table S1: Primers used in this study; Table S2: The proportions of sesquiterpenes (%) produced by wild type and variants of PentS; Figure S1: Schematic diagram of the expression constructs containing genes encoding the biosynthesis pathway of pentalenene (reference [14,45] is cited in supplementary materials); Figure S2: Mass spectra of main products produced by wild type and variants of PentS; Figure S3: Protein expression and solubility analysis of wild type and variants of PentS; Figure S4: Comparisons of peak patterns of T182 mutants; Figure S5: Multiple-sequence alignment of PentS (WP_186284259.1) with natural variants of pentalenene synthase; Figure S6: Binding pattern comparisons of PentS–FPP complex.
