# Supplementary material for: A Single Active-Site Mutagenesis Confers Enhanced Activity and/or Changed Product Distribution to a Pentalenene Synthase from Streptomyces sp. PSKA01
Source: Bioengineering (Basel). 2023 Mar 22;10(3):392. doi: 10.3390/bioengineering10030392 (PMC10045451; doi:10.3390/bioengineering10030392)
Supplement: Supplementary file 1 [file bioengineering-10-00392-s001.zip › bioengineering-2286601-supplementary.pdf]

bioengineering

MDPI

Article

A Single Active-Site Mutagenesis Confers Enhanced Activity and/or Changed Product Distribution to a Pentalenene Synthase from *Streptomyces* sp. PSKA01

Hongshuang Liu [1,2,†], Senbiao Fang [2,3,†], Lin Zhao [1,*], Xiao Men [2,3,*] and Haibo Zhang [2,3,*]

1 State Key Laboratory of Bio-Based Material and Green Papermaking, School of Bioengineering, Qilu University of Technology, Shandong Academy of Sciences, Jinan 250316, China
2 CAS Key Laboratory of Biobased Materials, Qingdao Institute of Bioenergy and Bioprocess Technology, Chinese Academy of Sciences, Qingdao 266101, China
3 Shandong Energy Institute, Qingdao 266101, China
* Correspondence: iahb205@163.com (L.Z.); menxiao@qibebt.ac.cn (X.M.); zhanghb@qibebt.ac.cn (H.Z.)
† These authors contributed equally to this work.

Supplementary Material

Table S1. Primers used in this study.

Name	Sequence (5′→3′)
PentS-F	TTAAGAAGGAGATATACCATGCCGCAGGATGTGGA
PentS-R	CCATCCAATTAACCTCCTTTAATGCGCGGTGCTACC
pE-F	AGGAGGTTAATTGGATGGACTTTCCGCAG
pE-R	GGTATATCTCCTTCTTAAAGTTAAAC
erPCR-F	TTAAGAAGGAGATATACCATGCCGCAGGATGTGGATTTTTA-TATTCCGCTGCCGG
erPCR-R	CCATCCAATTAACCTCCTTTAATGCGCGGTGCTAC-CTAATTCTTCTAAATAACCTTGCGCACC
Y150H-F	GCGTAATTATTTTAATGGCCATGTGGACGAGGCGGAGAGC
Y150H-R	GCTCTCCGCCTCGTCCACATGGCCATTAAAATAATTACGC
T182A-F	GGCGTGCAGCCGGCCGTTGATCTGG
T182A-R	CCAGATCAACGGCCGGCTGCACGCC
F192I-F	GGAACGTGCGGGTCGTATTGAAGTGCCACATCGC
F192I-R	GCGATGTGGCACTTCAATACGACCCGCACGTTCC
Q209R-F	CTGAGCGCGATGCTGCGGATTGCAGTTGATGTG
Q209R-R	CACATCAACTGCAATCCGCAGCATCGCGCTCAG
T182C-F	TGGCGTGCAGCCGTGCGTTGATCTGGCG
T182C-R	CGCCAGATCAACGCACGGCTGCACGCCA
T182D-F	TTGGCGTGCAGCCGGACGTTGATCTGGCGG
T182D-R	CCGCCAGATCAACGTCCGGCTGCACGCCAA
T182E-F	CATTGGCGTGCAGCCGGAGGTTGATCTGGCGGAAC
T182E-R	GTTCCGCCAGATCAACCTCCGGCTGCACGCCAATG
T182F-F	TTGGCGTGCAGCCGTTCGTTGATCTGGCGG
T183F-R	CCGCCAGATCAACGAACGGCTGCACGCCAA
T182G-F	TGGCGTGCAGCCGGGCGTTGATCTGGCG
T182G-R	CGCCAGATCAACGCCCGGCTGCACGCCA
T182H-F	TTGGCGTGCAGCCGCACGTTGATCTGGCGG
T182H-R	CCGCCAGATCAACGTGCGGCTGCACGCCAA
T182I-F	TTGGCGTGCAGCCGATCGTTGATCTGGC
T182I-R	GCCAGATCAACGATCGGCTGCACGCCAA

T182K-F	GGCGTGCAGCCGAAGGTTGATCTGGCGG
T182K-R	CCGCCAGATCAACCTTCGGCTGCACGCC
T182L-F	CCATTGGCGTGCAGCCGCTAGTTGATCTGGCGGAACG
T182L-R	CGTTCCGCCAGATCAACTAGCGGCTGCACGCCAATGG
T182M-F	GGCGTGCAGCCGATGGTTGATCTGGCGG
T182M-R	CCGCCAGATCAACCATCGGCTGCACGCC
T182N-F	TTGGCGTGCAGCCGAACGTTGATCTGGC
T182N-R	GCCAGATCAACGTTCGGCTGCACGCCAA
T182P-F	GGCGTGCAGCCGCCCGTTGATCTGG
T182P-R	CCAGATCAACGGGCGGCTGCACGCC
T182Q-F	CATTGGCGTGCAGCCGCAGGTTGATCTGGCGGAAC
T182Q-R	GTTCCGCCAGATCAACCTGCGGCTGCACGCCAATG
T182R-F	GGCGTGCAGCCGAGGGTTGATCTGGCGG
T182R-R	CCGCCAGATCAACCCTCGGCTGCACGCC
T182S-F	TGGCGTGCAGCCGAGCGTTGATCTGG
T182S-R	CCAGATCAACGCTCGGCTGCACGCCA
T182V-F	TTGGCGTGCAGCCGGTCGTTGATCTGGCGG
T182V-R	CCGCCAGATCAACGACCGGCTGCACGCCAA
T182W-F	CATTGGCGTGCAGCCGTGGGTTGATCTGGCGGAAC
T182W-R	GTTCCGCCAGATCAACCCACGGCTGCACGCCAATG
T182Y-F	CCATTGGCGTGCAGCCGTATGTTGATCTGGCGGAACG
T182Y-R	CGTTCCGCCAGATCAACATACGGCTGCACGCCAATGG

Table S2. The proportions of sesquiterpenes (%) produced by wild type and variants of PentS. *

	a**	1	2	3	4	5	b**	6
PentS-WT	0.51±0.01	98.28±2.7	N.D.	N.D.	N.D.	0.29±0.08	0.21±0.03	0.71±0.10
T182A	2.47±0.03	60.11±3.00	6.85±0.62	3.16±0.03	0.20±0.03	2.13±0.11	17.93±0.95	7.14±0.55
T182C	1.41±0.15	89.78±13.21	N.D.	1.16±0.21	N.D.	0.84±0.15	1.35±0.25	5.47±0.87
T182I	N.D.	68.46±10.21	N.D.	2.36±0.89	2.79±0.59	3.62±0.39	6.08±0.82	16.69±1.40
T182S	1.00±0.05	96.55±8.89	N.D.	0.18±0.21	N.D.	0.30±0.08	0.98±0.06	0.98±0.07
T182V	0.55±0.02	82.89±4.61	N.D.	1.67±0.65	3.41±0.26	2.30±0.17	5.05±0.37	4.13±0.35

* The completely inactivating mutations are not shown in the table.
** **a** and **b** indicate unidentified hydrocarbons with m/z 204. **1**, pentalenene; **2**, sativene; **3**, longifolene-V4; **4**, β-chamigrene; **5**, thujopsene-I3; **6**, β-elemene. N.D., not detectable.

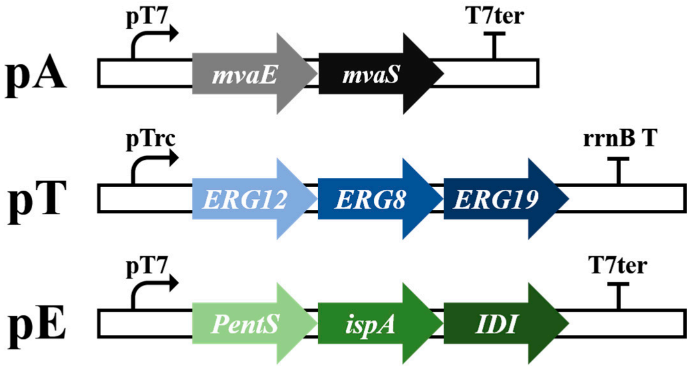

Figure S1. Schematic diagram of the expression constructs containing genes encoding the biosynthetic pathway of pentalenene [1,2]. *mvaE*, acetoacetyl-CoA synthase/HMG-CoA reductase; *mvaS*, HMG-CoA synthase; *ERG12*, mevalonate kinase; *ERG8*, phosphomevalonate kinase; *ERG19*, mevalonate pyrophosphate decarboxylase; *PentS*, pentalenene synthase; *ispA*, farnesyl pyrophosphate (FPP) synthase; *IDI*, isopentenyl pyrophosphate (IPP) isomerase.

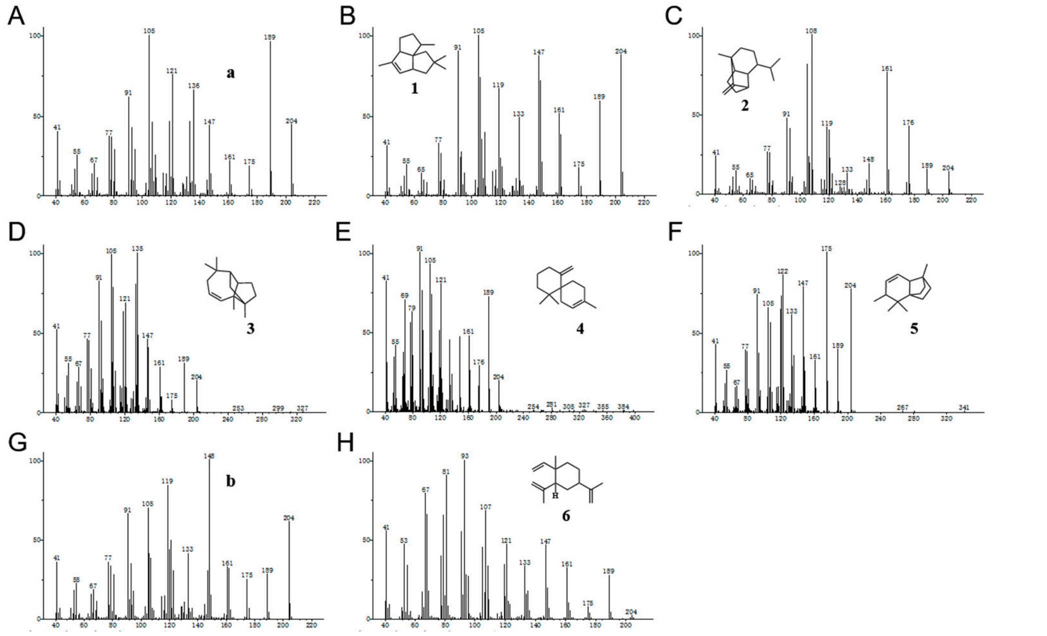

Figure S2. Mass spectra of main products produced by wild type and variants of PentS. **a** and **b** indicated the mass spectra of unidentified hydrocarbons, m/z 204, **1**-**6** were the mass spectra of identified substances, respectively pentalenene, sativene, longifolene-V4, β-chamigrene, thujopsene-I3 and β-elemene.

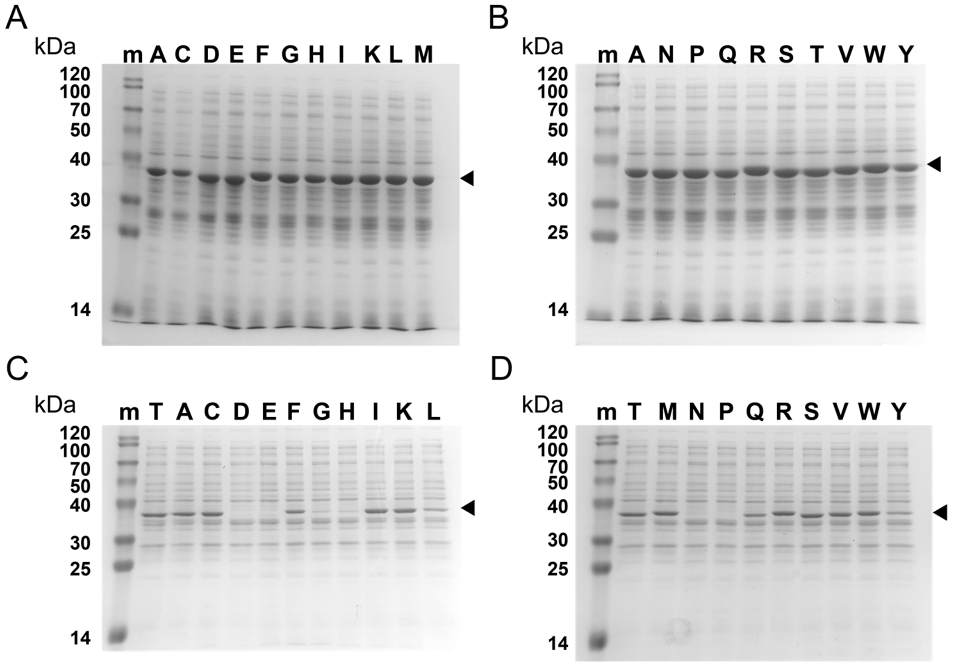

Figure S3. Protein expression and solubility analysis of wild type and variants of PentS. (A) and (B) Expression levels of T182 saturated mutants in E. coli BL21(DE3); (C) and (D) Soluble fractions of the T182 saturated mutants. m, protein marker; the 20 capital letters represented the abbreviations of 20 amino acids.

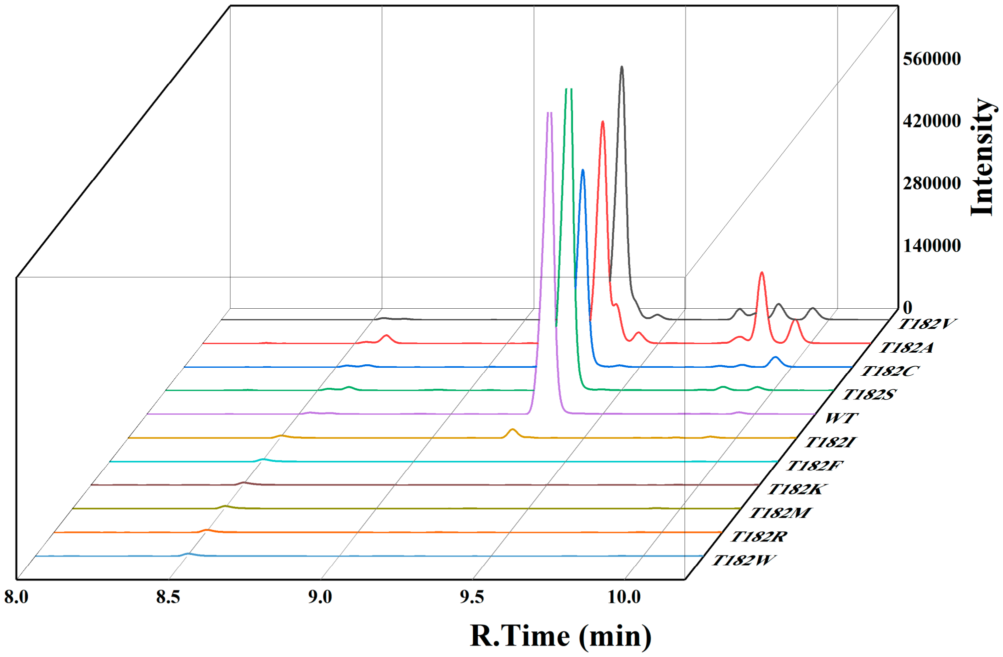

Figure S4. Comparison of peak patterns of T182 mutants.

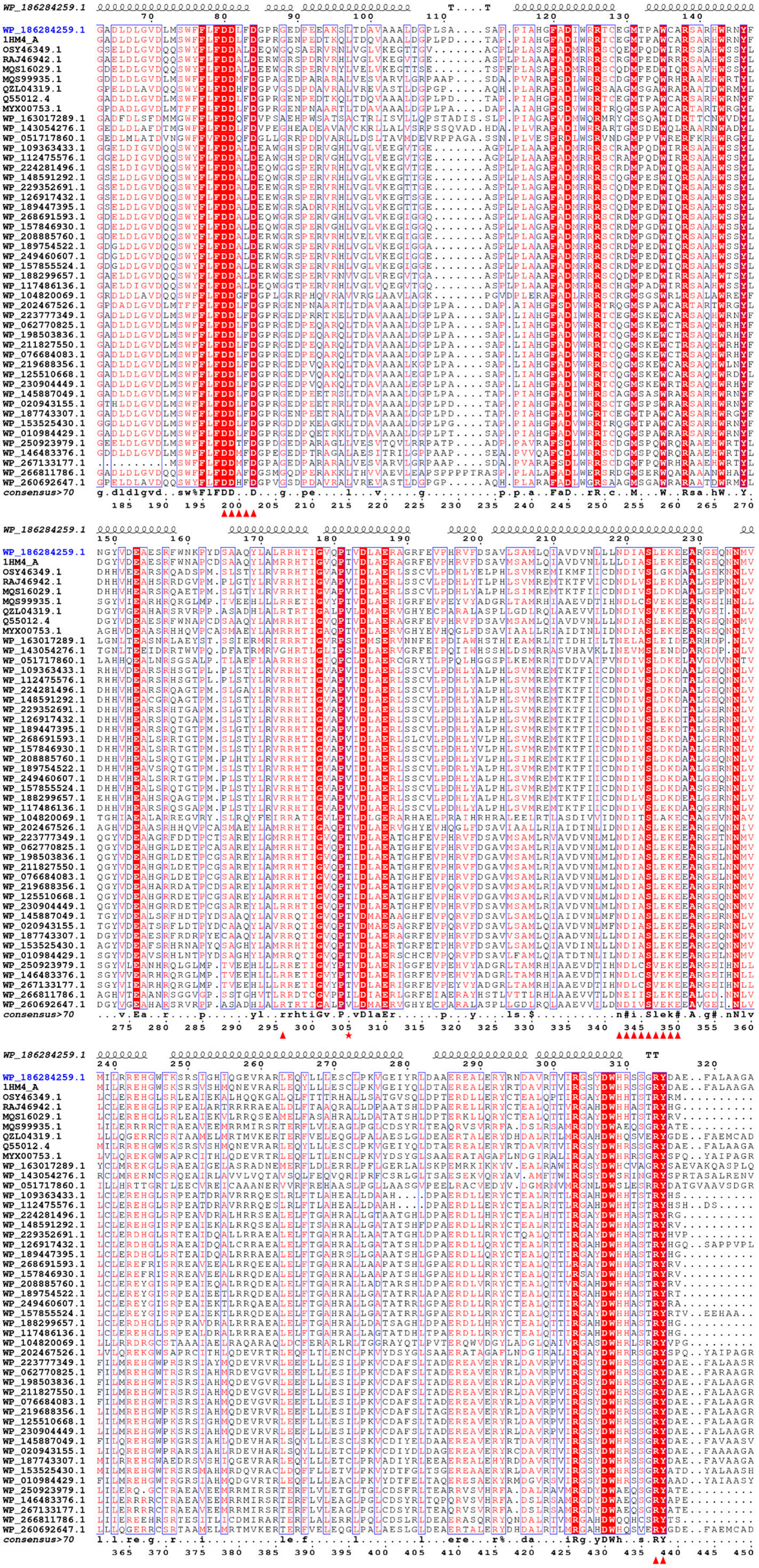

Figure S5. Multiple sequence alignment of PentS (WP_186284259.1) with natural variants of pentalenene synthase. The red triangles represented the conserved "^{80}DD××D/E", "^{173}R", "^{219}NSE/DTE" and "^{314}RY" motifs. The red asterisk represented T182 position.

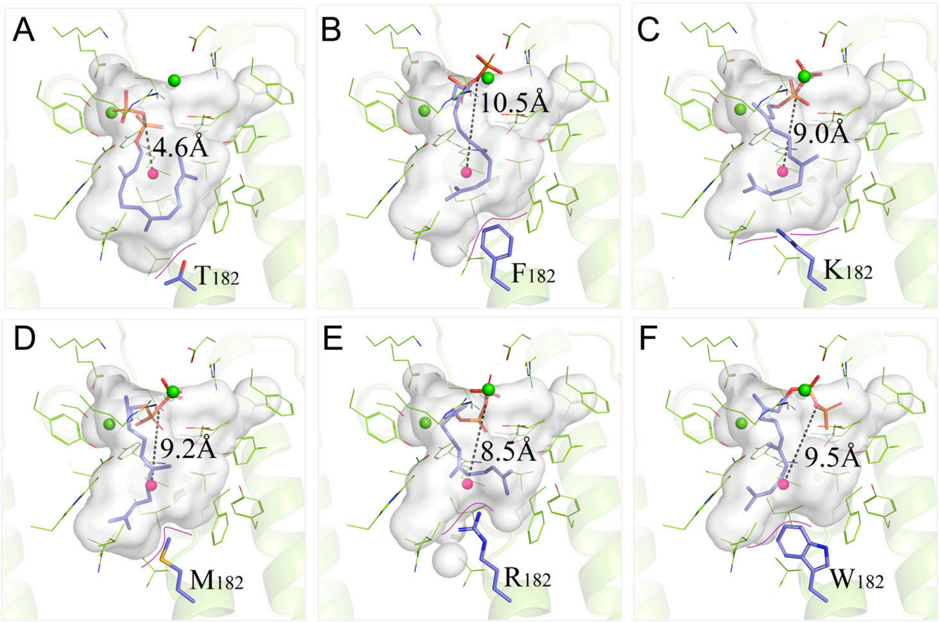

Figure S6. Binding pattern comparisons of PentS-FPP complexes. The secondary structures of wild type and variants of PentS were shown as cartoon and colored in limon. The binding cavities were outlined by white surfaces. The residues at position 182 were shown as sticks in which atoms were color-coded as follows: C, blue; N, dark blue; O, red; S, yellow. The geometric center of the binding cavity of the wild-type PentS (red sphere) was used as a comparing template , and the $D_{O\text{-}C}$ is marked with black dashed lines.

References

1. Zhang, H.; Liu, Q.; Cao, Y.; Feng, X.; Zheng, Y.; Zou, H.; Liu, H.; Yang, J.; Xian, M. Microbial production of sabinene—a new terpene-based precursor of advanced biofuel. *Microb. Cell Fact.* **2014**, *13*, 20, doi:10.1186/1475-2859-13-20.
2. Yang, J.; Xian, M.; Su, S.; Zhao, G.; Nie, Q.; Jiang, X.; Zheng, Y.; Liu, W. Enhancing production of bio-isoprene using hybrid MVA pathway and isoprene synthase in *E. coli*. *PLoS One* **2012**, *7*, e33509, doi:10.1371/journal.pone.0033509.
